# AI-Supported Digital Microscopy Diagnostics in Primary Health Care Laboratories: Scoping Review

**DOI:** 10.2196/78500

**Published:** 2026-01-05

**Authors:** Joar von Bahr, Antti Suutala, Vinod Diwan, Andreas Mårtensson, Johan Lundin, Nina Linder

**Affiliations:** 1 Department of Global Public Health, Karolinska Institutet, Tomtebodavägen 18 A, Solna, 17165, Sweden, 46 708561007; 2 Global Health and Migration Unit, Department of Women’s and Children’s Health, Uppsala University, Uppsala, Sweden; 3 Institute for Molecular Medicine Finland (FIMM), HiLIFE, University of Helsinki, Helsinki, Finland; 4 Department of Infectious Diseases, Uppsala University Hospital, Uppsala, Sweden

**Keywords:** AI, artificial intelligence, convolutional neural network, deep learning, diagnosis, digital diagnostics, machine learning, pathology, primary health care, whole slide images

## Abstract

**Background:**

Digital microscopy combined with artificial intelligence (AI) is increasingly being implemented in health care, predominantly in advanced laboratory settings. However, AI-supported digital microscopy could be especially advantageous in primary health care settings, since such methods could improve access to diagnostics via automation and a decreased need for experts on-site. To our knowledge, no scoping or systematic review has previously examined the use of AI-supported digital microscopy in primary health care laboratories, and a scoping review could guide future research by providing insights into the challenges of implementing these novel methods.

**Objective:**

This scoping review aimed to map published peer-reviewed studies on AI-supported digital microscopy in primary health care laboratories to generate an overview of the subject.

**Methods:**

A systematic search of the databases PubMed, Web of Science, Embase, and IEEE was conducted on October 2, 2024. The inclusion criteria in the scoping review were based on 3 concepts: using digital microscopy, AI, and comparison of the results with a standard diagnostic system, and 1 context, being performed in primary health care laboratories. Additional inclusion criteria were peer-reviewed diagnostic accuracy studies published in English, performed on humans and achieving a sample-level diagnosis. The study selection and data extraction were performed by 2 independent researchers (JVB and AS), and cases of disagreement were resolved through discussion with a third researcher (NL). The methodology is in accordance with the Joanna Briggs Institute methodology for scoping reviews.

**Results:**

A total of 3403 papers were screened during the paper identification process, of which 22 (0.6%) were included in the scoping review. The samples analyzed were as follows: blood (n=12) for blood cell and malaria detection, urine (n=4) for urinalysis and parasite detection, cytology of atypical oral (n=1) and cervical cells (n=2), stool (n=2) for parasite detection, and sputum (n=1) for ferning patterns indicating inflammation. Both conventional (n=15) and specifically developed methods (n=7) were used in sample preparation. The AI-supported digital microscopy achieved comparable diagnostic accuracy to the reference standard for complete blood counts, malaria detection, identification of stool and genitourinary parasites, screening for oral and cervical cellular atypia, detection of pulmonary inflammation, and urinalysis. Furthermore, AI-supported digital microscopy achieved higher sensitivity than manual microscopy in 6/7 (85.7%) studies that used a reference standard that allowed for this comparison.

**Conclusions:**

AI-supported digital microscopy achieved comparable diagnostic accuracy to the reference standard for diagnosing multiple targets in primary health care laboratories and may be particularly advantageous for improving diagnostic sensitivity. With further research addressing challenges such as scalability and cost-effectiveness, AI-supported digital microscopy could improve access to diagnostics, especially in expert-scarce and resource-limited settings.

## Introduction

### Background

Artificial intelligence (AI) in the form of machine learning has successfully been applied to image-based diagnostics within several medical fields [1]. In parallel, manual microscopy remains a cornerstone of diagnostic practice in resource-limited settings and at the primary health care (PHC) level due to its low cost, versatility, and ability to provide direct visualization of pathogens and cellular changes. It is widely used for the diagnosis of infectious diseases such as malaria and intestinal parasitic infections, as well as for full blood counts and analysis of cervical and oral cytological samples and fine needle aspirates [2]. Despite its usefulness and broad applicability, microscopy is highly dependent on the availability of trained personnel and adequate infrastructure, which are often limited in such settings, leading to variability in diagnostic quality and coverage [3]. These limitations have motivated the development of AI-driven approaches, where deep learning methods can assist or automate microscopy-based diagnostics to improve accuracy and accessibility. Deep learning approaches, particularly convolutional neural networks (CNNs) and vision transformers, have become the dominant architectures for image classification and interpretation in medical imaging [4]. CNNs extract visual features, enabling recognition of complex structures such as cells, pathogens, and tissue patterns, while vision transformers can capture contextual relationships between distant structures [4,5].

Leveraging these methods for AI-based microscopy within laboratory workflows has the potential to automate processes, increase productivity, and improve diagnostic accuracy [6]. Multiple AI-based diagnostic systems have been approved for clinical use, for example, for cervical cancer screening and prostate cancer diagnostics [6-8]. Most of these AI-based diagnostic systems depend on expensive, high-end digital imaging instruments and require advanced laboratory infrastructure and are therefore not feasible for use in PHC laboratories [6,7]. However, the development of less expensive, portable digital microscope scanners has enabled research on the use of AI-supported diagnostic systems suitable for PHC laboratories [9-11].

A PHC laboratory, also known as a tier 1 laboratory, can be defined as a laboratory primarily serving outpatients by providing point-of-care (POC) tests and manual microscopy of specimens with simple preparations. An additional responsibility is preparing fine needle aspirations and other simple tissue specimens that are later dispatched to a tier 2 laboratory in a first-level hospital for analysis. The PHC laboratories work with a small budget compared with more advanced laboratories and are generally managed by a laboratory technician supervised by a pathologist from a distance [2].

The World Health Organization has emphasized the importance of providing diagnostics near the patient to enhance the accuracy and timeliness of diagnoses, improve clinical decision-making, and reduce the risk of diagnostic errors [12]. The implementation of AI-supported digital microscopy could help address these challenges at PHC laboratories. To begin with, since PHC laboratories lack access to pathology expertise, application of AI could enable more analyses on-site, consequently increasing both the availability and speed of diagnostics [2,13]. Increased speed and access to diagnostics through AI and telemedicine could reduce health inequities by strengthening diagnostic capacity, particularly in low- and middle-income countries (LMICs) and also in sparsely populated regions of high-income countries [11,14,15]. In addition, a systematic review showed that the implementation of AI-supported diagnostics for microscopy increased the effectiveness of laboratory personnel [6]. Although there is a global shortage of microscopy experts, the shortage of these specialists is more severe in LMICs; therefore, AI-supported digital microscopy may be especially advantageous in strengthening health systems and reducing the diagnostic gaps in these settings [11,16].

There are several diseases where AI-supported digital microscopy diagnostics in PHC laboratories could be advantageous, and studies have been performed on, for example, screening of oral and cervical cancer as well as targeting parasitic infections, such as schistosomiasis and infections caused by soil-transmitted helminths [9,17-19]. Although the targeted diseases differed in these studies, researchers often encountered similar challenges due to commonalities in the methodologies applied, and a review mapping these challenges could provide valuable insights.

A preliminary search of the databases PubMed and Cochrane was performed to investigate whether any scoping or systematic review had been performed on AI-supported digital microscopy in PHC laboratories. A few related reviews were found. One systematic review of AI diagnostics for oral cancer [20] overlaps to some extent with our review; however, since it focuses on a single disease, it does not provide an overview of the development of AI-supported digital microscopy in PHC laboratories. Another systematic review evaluating the application of AI to whole slide images of tissue samples stained with hematoxylin and eosin was also identified [21]. This paper presents the current state of knowledge on AI implementation in pathology within high-end laboratories.

While these reviews are similar to this scoping review, they do not provide an overview of which diseases have been investigated in AI-supported digital microscopy and the disease-agnostic challenges faced in PHC laboratories. Furthermore, the development of more affordable scanners and improved AI, along with persistent workforce and resource constraints, makes a scoping review timely. A scoping review performed on AI-supported digital microscopy in PHC laboratories would, therefore, provide a valuable overview of the subject and collate knowledge that could guide future implementation.

This scoping review aimed to systematically review published peer-reviewed studies that have been performed related to AI-supported digital microscopy in PHC laboratories and specifically address the following questions: (1) In which diseases and for which conditions and targets has AI-based microscopy been applied for diagnostics within PHC laboratories? (2) What methods have been used in acquiring microscopy images to train and analyze AI models for diagnostics? (3) What AI models and training approaches have been applied? (4) How has the AI-supported diagnostic system performed compared with expert microscopists with regard to diagnostic accuracy?

### Review Question

What peer-reviewed studies have been published on implementing AI-supported digital microscopy in PHC laboratories? What methods have been used, what issues have been faced, and what results have been achieved?

## Methods

### Study Design

The scoping review was conducted in accordance with the Joanna Briggs Institute methodology for scoping reviews updated in 2020 [22]. A PRISMA-ScR (Preferred Reporting Items for Systematic reviews and Meta-Analyses extension for Scoping Reviews) checklist is included [23]. A protocol was initially published in the Open Science Framework and later in the peer-reviewed journal JMIR Research Protocols [24,25]. The inclusion and exclusion criteria are shown in Table 1.

**Table 1.** Inclusion and exclusion criteria for identified studies.

Study characteristic	Inclusion criteria	Exclusion criteria
Language	English	Non-English
Study design	Published peer-reviewed studies Diagnostic test accuracy studies	Non–peer reviewed studies Not diagnostic test accuracy studies
Population	Humans	Studies performed on animals
Concept	AI^a^ techniques applied as a diagnostic tool on microscopy Final slide-level diagnosis was performed and compared with a standard microscopist Outcome valuable for clinicians	Studies that applied AI models on images not conventionally analyzed in microscopy No final slide diagnosis
Context	Performed at primary health care laboratory (tier 1 laboratory) No pathologist needed on site Samples such as stool, urine, blood, cytology smears, and fine needle aspirations of superficial tissue (eg, from breast lumps) prepared with simple methods	Studies performed in an advanced laboratory setting

a AI: artificial intelligence.

### Eligibility Criteria

#### Participants

This scoping review considered studies on human participants. No exclusion was made based on age, sex, economic status, or nationality.

#### Concept

The studies included in this scoping review fulfilled 3 concept criteria. First, the studies needed to be performed on images obtained with an imaging instrument built to automatically capture microscopy sample areas large enough for diagnostic purposes. Furthermore, the imaging instrument used must be operated in a way that does not require human expertise to determine what areas of the slide should be captured. Microscopy was defined as deploying a light source, optical lenses, and a digital camera to acquire a magnified image of a biological sample, generating an image conventionally interpreted by a microscopist.

Second, the studies needed to use AI when analyzing the microscopy images. AI was defined as a computer system that is trained to perform a task that typically requires human intelligence. No exclusion was made based on the architecture of the AI model or the dataset used for training. This analysis of the microscopy images could be performed on-site or in a remote cloud environment.

Third, the studies needed to compare the AI-supported diagnostic system with a standard diagnostic system. A diagnostic system was defined as all the steps included in the diagnostic process, from sample collection to the acquisition of results. The result needed to be sufficient to reach a diagnosis at the subject level.

#### Context

The included studies needed to be performed in a PHC laboratory setting. To be defined as a PHC laboratory, also known as a tier 1 laboratory, the laboratory needed to fulfill 2 criteria. First, regarding staffing, the laboratory must be run by a laboratory technician, not requiring a pathologist on-site. Second, the sample preparations could not exceed the capabilities of a PHC laboratory. Acceptable samples collected included stool, urine, blood, cytology smears, and fine needle aspirations of superficial and easily accessible tissues (eg, from breast lumps and superficial lymph nodes). The sample staining procedure must be possible to perform manually without advanced laboratory equipment such as a microtome or tissue processor [2]. Sample procedures that fulfill these criteria include Kato-Katz thick stool smears, blood smears, centrifuged urine samples, Papanicolaou-stained cervical or oral smears, and hematoxylin and eosin–stained fine needle cytology smears [2]. Since the context of PHC laboratories in this scoping review is based on human medicine, the exclusion criteria and initial search strategy were changed to exclude veterinary medicine, which was included in the initial protocol published on Open Science Framework [24]. This adjustment was made before submitting the protocol to JMIR Research Protocols to focus the scoping review specifically on challenges in implementing AI-supported microscopy in human health care [25].

### Types of Sources

All types of diagnostic test accuracy studies were included. Because data collection in diagnostic test accuracy studies can be both retrospective and prospective, studies using either approach were included. In addition, studies using both paired and random designs for reference standards were included [26]. The included studies had to be published in English.

### Search Strategy

The search strategy was designed to identify peer-reviewed published papers. An initial limited search of PubMed and Cochrane was undertaken to identify papers on the topic. Search blocks were created for the final search based on terms used in the identified papers. The search blocks were developed to find papers containing the 2 concepts, microscopy and AI, as well as the context specification of being in a PHC setting, with 1 block created for each. The databases searched were PubMed, Web of Science, Embase, and IEEE, and a detailed description of the search strategy is given in Multimedia Appendix 1. The search was performed on October 2, 2024. The reference lists and all the papers citing the included papers were gathered through the SpiderCite tool on December 3, 2024, and included in the review process [27].

### Study Selection

Following the search, all identified papers were compiled in a reference management software system, Zotero (version 6.0.20, Digital Scholar; January 13, 2023, opensource) and duplicates removed. Following the pilot test, titles and abstracts were screened by 2 independent reviewers (JvB and AS) for assessment against the inclusion and exclusion criteria using Covidence systematic review software (Veritas Health Innovation, 2024) [28]. During this step, the Cohen κ agreement was 0.59. All disagreements between JvB and AS were resolved by NL, who provided the deciding vote and could consult the other screeners for their rationale. Thereafter, the full texts of the remaining papers were assessed in detail against the inclusion criteria by 2 independent reviewers (JvB and AS). During full text screening, the Cohen κ agreement was 0.75 for the database search and 0.36 for the citation search. Two reasons caused 17 out of 21 disagreements in the citation search and were resolved through discussions between JvB, AS, JL, and NL. The first issue concerned whether urine analyzers such as Iris iQ200 or Sysmex UF-100 fulfilled the PHC criteria: it was concluded that they did not, as these devices perform advanced sample preprocessing within the machine [29]. The second issue concerned whether handcrafted feature classification qualified as AI: it was concluded that it did not, as it does not involve AI training. With these 2 issues resolved, the citation search had a Cohen κ agreement of 0.79.

### Data Charting and Synthesis

Data were extracted from the studies included in the scoping review by 2 reviewers (JvB and AS) using a data extraction tool developed with Covidence systematic review software. The predeveloped extraction tool can be found in Multimedia Appendix 2. Initially, the extraction was performed by JvB. Afterward, the extracted information was checked by AS. All disagreements were resolved through discussion between JvB and AS. When questions arose regarding an original paper, the corresponding author of that manuscript was contacted. The findings are presented narratively and additionally in a table format based on the extraction tool. The information from the extraction tool was split into 3 tables and 1 figure to increase readability. The figure contains a simplified overview of the studies, the first table summarizes the process from sample collection to scanning, the second table shows information on the AI analysis pipeline and training data, and the third table reports the study outcomes. Based on the information extracted to the tables, a narrative description was written to provide an overview of the mapped information. The studies were grouped based on the sample type investigated and the disease targeted as per the first objective of the study: to map in which diseases and for which conditions and targets has AI-based microscopy been applied for diagnostics within PHC laboratories.

### Critical Appraisal of Results

The QUADAS-2 tool was applied to investigate the bias of the included studies. This tool was developed to assess the risk of bias for diagnostic accuracy studies in 4 areas: patient selection, index test, reference standard, and flow and timing [30]. The results are shown in the “Results” section, and the form used can be found in Multimedia Appendix 3.

## Results

### Overview

**Figure 1.**
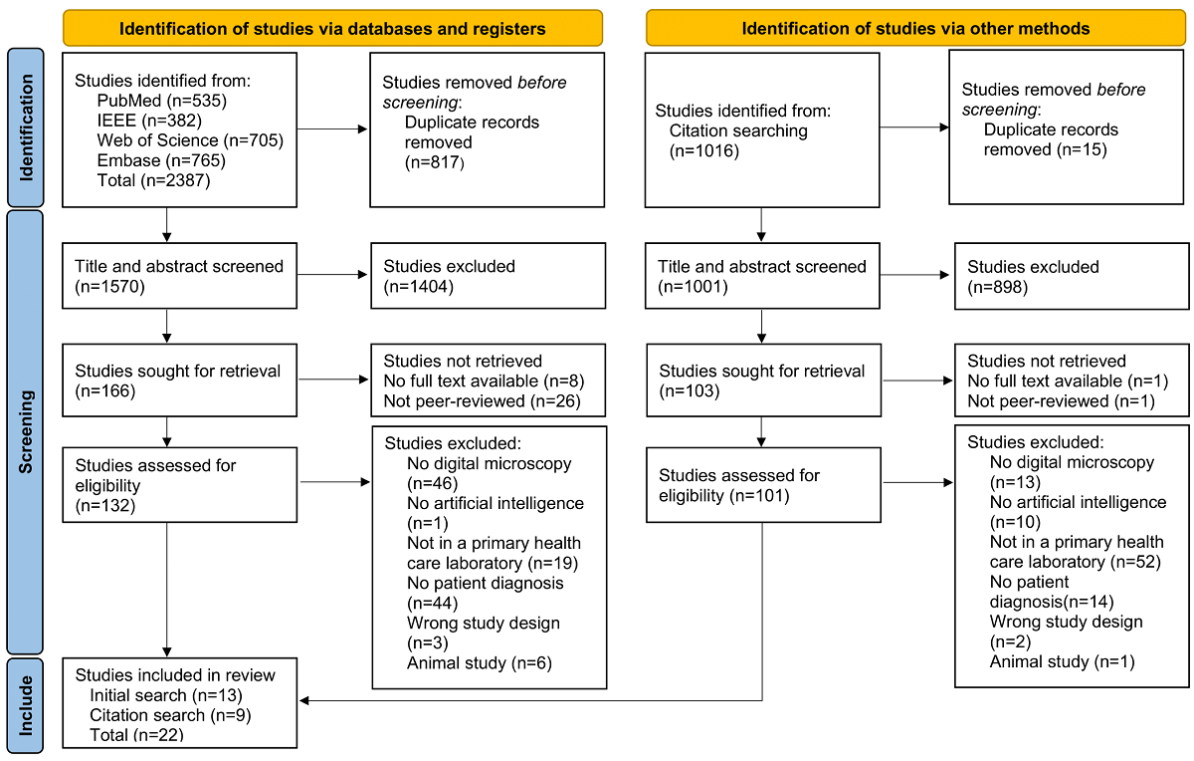
Flowchart for study inclusion.

In total, 3403 papers were screened during the paper identification process, of which 22 (0.6%) were included in the scoping review. The results of the search and the study inclusion process are reported in full in a PRISMA (Preferred Reporting Items for Systematic reviews and Meta-Analyses) flow diagram (Figure 1) [31].

**Figure 2.**
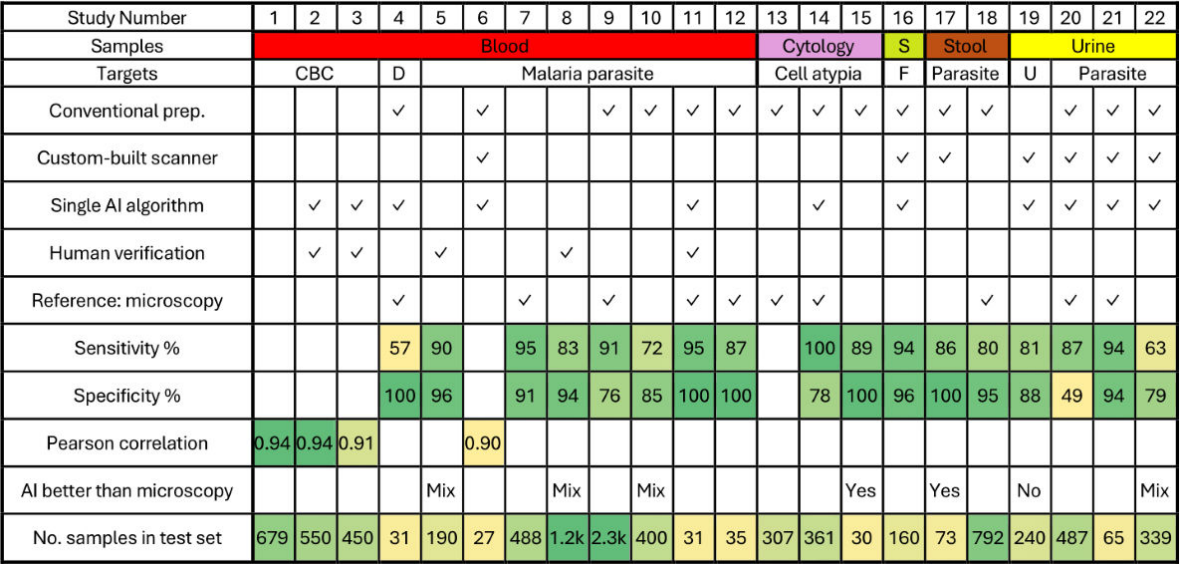
Overview of the included studies. AI better than microscopy: White = No comparison, Yes = Higher/same sensitivity and specificity, Mix = Higher sensitivity and lower specificity, and No = Lower sensitivity and specificity. Number of samples in the test set: k=1000. Conditional formatting was applied to all numerical values, with high values shaded green and low values shaded yellow. AI: artificial intelligence; CBC: complete blood count; D: Downey cells; F: Ferning patterns indicative of inflammation; S: sputum; U: urinalysis.

The oldest included study was published in 2014, while the remaining studies were published in 2018 or later, with 9 out of the 22 (40.9%) studies published in 2024. The papers were published in 15 different journals with the most common being *Malaria Journal* (n=4) and *PLOS One* (n=4). The most analyzed samples were blood (n=12), followed by urine (n=4), cytology (n=3), stool (n=2), and sputum (n=1). Different parasites (malaria, intestinal, or genitourinary parasites) were the most common targets (n=13), followed by blood cells (n=4), atypical cervical cells (n=2), atypical oral cells (n=1), and urine particles, such as cells (n=1) and crystalline ferning patterns in sputum (n=1). Detection of these targets was used for multiple diseases and conditions; complete blood counts (CBCs) and urinalysis were used for both organ-specific and systemic diseases, parasite detection for corresponding infections, atypical cells for screening and detection of cancer, and ferning patterns for identifying pulmonary inflammation in patients with COVID-19. An overview of all studies is shown in Figure 2.

### Sample Preparation and Scanning

Out of the 22 included studies, 12 relied solely on manual preparation methods and 3 used centrifuges. The remaining studies (n=7) used cartridges that simplified and eliminated manual steps. Both in-house–built and commercially available scanners such as Grundium, MiLab, and Motic EasyScan GO were used. The lowest numerical aperture used was 0.1 and the highest was 1.4. Several scanners used both autofocus algorithms and z-stacking to avoid out-of-focus areas. In the 5 studies reporting the time from sample collection to diagnosis using AI-supported digital microscopy, it was 20‐40 minutes, but there was also a study reporting that it took more than 50 minutes for the scanning and AI analysis (Table 2) and a more detailed table in Multimedia Appendix 4.

**Table 2.** Time for analysis and sample processing for the included studies.

Study	Sample	Target	Sample preparation	Sample scanning	Time for analysis
Bachar et al (2021) [32]	Blood	CBC^a^	Cartridge with 2 stains	No retrievable magnification and resolution	No retrievable information
Gasparin et al (2023) [33]	Blood	CBC	Dual-chamber cartridge with 2 stains	No retrievable magnification and resolution	Total: 30‐40 minutes
Gasparin et al (2022) [34]	Blood	CBC	Dual-chamber cartridge with 2 stains	No retrievable magnification and resolution	Total: 30‐40 minutes
Akisin et al (2023) [35]	Blood	Downey cells	Manual blood smears stained with May-Grünwald and Giemsa	100× with oil immersion	No retrievable information
Hamid et al (2024) [36]	Blood	Malaria parasites	Cartridge with Giemsa staining	A resolution similar to 50× microscopy [37]	Total: <30 minutes
Holmström et al (2020) [38]	Blood	Malaria parasites	Manual blood smears stained with DAPI^b^	A resolution of 0.9 µm	No retrievable information
Bae et al (2024) [37]	Blood	Malaria parasites	Cartridge with Giemsa staining	A resolution similar to 50×	Total: <30 minutes [36,39]; Scanning: 7‐10 minutes
Ewnetu et al (2024) [39]	Blood	Malaria parasites	Cartridge with Giemsa staining	A resolution similar to 50× [37]	Total: circa 20 minutes
Das et al (2022) [40]	Blood	Malaria parasites	Manual blood smears stained with Giemsa	40× (NA^c^ 0.75)	Scanning and AI^d^ analysis: 20‐30 minutes
Torres et al (2018) [41]	Blood	Malaria parasites	Manual blood smears stained with Giemsa	100× with oil immersion (NA 1.25)	No retrievable information
Linder et al (2014) [42]	Blood	Malaria parasites	Thin blood smears stained with Giemsa	63× with oil immersion (NA 1.4)	No retrievable information
Horning et al (2021) [43]	Blood	Malaria parasites	Manual blood smears stained with Giemsa	40x (NA 0.75)	Scanning and AI analysis 54 minutes
Stegmüller et al (2024) [44]	Cervical cytology	Cellular atypia	SurePath procedure with Papanicolaou stain	40× (NA 0.75)	No retrievable information
Holmström et al (2021) [9]	Cervical cytology	Cellular atypia	Conventional Papanicolaou smears	20× (NA 0.4)	Scanning: 5‐10 minutes; uploading 10‐40 minutes
Sunny et al (2019) [19]	Oral cytology	Cellular atypia	Manual liquid-based cytology, stained with H&E^e^ [45]	20× (NA 0.4) [45]	AI analysis: 10 minutes
Ghaderinia et al (2024) [46]	Sputum	Ferning patterns	Sedimented unstained sputum samples	40× magnification	No retrievable information
Soares et al (2024) [47]	Stool	Intestinal parasites	Fecal samples with centrifugation, flotation, and sedimentation [48]	No retrievable magnification and resolution	AI analysis: circa 3 minutes
Lundin et al (2024) [49]	Stool	Soil-transmitted helminths	Kato-Katz thick smears	20× (NA 0.4)	Scanning 5‐10; uploading 10‐20 minutes; AI analysis 5 minutes
Sahu et al (2024) [50]	Urine	Urinalysis	Cartridge that concentrates the urine through 5 minutes of sedimentation	40× (NA 0.65)	No retrievable information
Meulah et al (2022) [51]	Urine	Schistosoma	A membrane capturing filtered urine particles	4× (NA 0.1)	Scanning: 12 minutes; AI analysis: 5 minutes
Oyibo et al (2022) [52]	Urine	Schistosoma	A membrane capturing filtered urine particles	4× (NA 0.1)	Scanning: 12 minutes; AI analysis: 10‐12 minutes
Meulah et al (2024) [53]	Urine	Schistosoma	A membrane capturing filtered urine particles	4× (NA 0.1)	Scanning and AI analysis: 25 minutes

a CBC: complete blood count.

b DAPI: 4',6-diamidino-2-phenylindole.

c NA: numerical aperture.

d AI: artificial intelligence.

e H&E: hematoxylin and eosin.

### Training Data and AI Analysis Pipeline

For training AI models, most studies used in-house collected and annotated datasets of varying sizes; some had hundreds of target objects in their dataset, whereas others had hundreds of thousands. Many studies reported using pretrained neural networks with different datasets such as COCOtrain2017 and ImageNet for training [47,53]. One study used unlabeled data from their collection for unsupervised pretraining and incorporated publicly available datasets [44].

**Figure 3.**
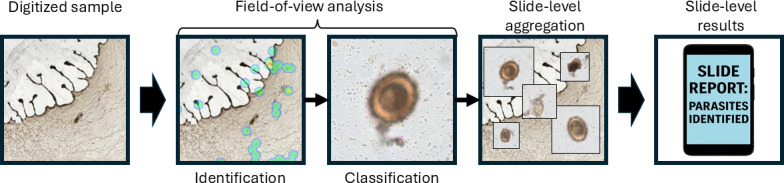
Visualization of the artificial intelligence analysis pipeline. As an illustrative case, the pipeline is applied to a digitized fecal smear with *Ascaris lumbricoides* parasite eggs.

The AI analysis pipeline for all included studies can be summarized as follows: a digitized microscopy sample was provided as input, fields-of-view (FOVs) were analyzed, FOV results were aggregated to produce a slide-level diagnosis, and this diagnosis served as the output (Figure 3). The digitized sample used as input could consist of either whole-slide images or multiple FOVs captured from the physical slide. The FOV analysis involved both the identification and the classification of specific targets; however, not all studies used this first identification of suspicious FOVs (regions of interest).

An initial detection of suspicious FOVs was described in half of the studies; when this was applied, algorithmic approaches, shallow CNNs, or support vector machines (SVMs) were used. The purpose of performing this initial identification of FOVs of interest was to reduce the number of FOVs that needed to be analyzed by more computationally expensive AI algorithms. An additional advantage of performing an initial detection of suspicious FOVs was that the FOVs were more homogeneous in content and quality, which can improve the accuracy of the AI classifier. All studies used AI for the FOV classification step, predominantly CNNs, except for the oldest study that used an SVM [42]. One approach was to use multiple classification steps, for example, by using SVM or shallow CNNs to classify targets and then reclassifying those with higher uncertainty with deeper CNNs [37,47].

To achieve the slide-level diagnosis from the FOV analysis results, multiple methods were used; for example, classifying slides with any number of positive targets as positive, using different cutoffs based on confidence or number of findings, or using AI-based methods such as SVMs and multiple instance learning (Table 3).

**Table 3.** Artificial intelligence model training and architecture for the included studies.

Study	Sample and target	Samples in training set	AI^a^ model architecture and training
Bachar et al (2021) [32]	Blood and CBC^b^	No retrievable information	AI model with separate pipelines for platelets, RBCs^c^, and WBCs^d^ (1) algorithmically identifies candidates, and (2) candidates categorized by specialized CNNs^e^ and machine learning algorithms
Gasparin et al (2023) [33]	Blood and CBC	Expert-verified training data gathered throughout development; no further retrievable information	AI model with CNN architecture using the YOLO^f^ framework
Gasparin et al (2022) [34]	Blood and CBC	Expert-verified training data gathered throughout development; no further retrievable information [33]	AI model with CNN architecture using the YOLO framework
Akisin et al (2023) [35]	Blood and Downey cells	15,885 expert-annotated WBCs containing 172 Downey cells	AI model with YOLOv4-tiny-based framework with spatial attention using average and maximum pooling along the channel axis
Hamid et al (2024) [36]	Blood and malaria parasites	No retrievable information	AI model with (1) U-Net segmenting RBCs, (2) a 3-layer CNN removing normal RBCs, (3) a 23-layer CNN for detecting parasites, and (4) 1 positive object sufficient for slide positivity [37]
Holmström et al (2020) [38]	Blood and malaria parasites	25 thin blood smears with annotated trophozoites (n=5059) and other fluorescence signals (n=856)	AI model with (1) Circle Hough Transform identifying RBCs, (2) fluorescence signals from within the detected RBCs are used, and (3) RBCs with fluorescence signals were analyzed with a CNN (GoogLeNet)
Bae et al (2024) [37]	Blood and malaria parasites	No retrievable information	AI model with (1) U-Net segmenting RBCs, (2) a 3-layer CNN removing normal RBCs, (3) a 23-layer CNN for detecting parasites, and (4) 1 positive object sufficient for slide positivity
Ewnetu et al (2024) [39]	Blood and malaria parasites	No retrievable information	AI model with (1) U-Net segmenting RBCs, (2) a 3-layer CNN removing normal RBCs, (3) a 23-layer CNN for detecting parasites, and (4) 1 positive object sufficient for slide positivity [37]
Das et al (2022) [40]	Blood and malaria parasites	Subset of 1452 blood samples and 956,531 annotated parasite objects [54]	AI model analyzes only thick region with (1) potential parasites identified through dynamic thresholding and SVM^g^, (2) CNN (VGG^h^ architecture) classifies parasites, and (3) a predetermined threshold decides slide positivity [54]
Torres et al (2018) [41]	Blood and malaria parasites	Approximately 150 high-quality thick films with 75,000 parasites	AI model for thick region with (1) local thresholding and low-cost methods to identify potential parasites, (2) CNNs (VGG architecture) classifies parasites and stage, and (3) number and confidence of parasites determine slide-level diagnosis
Linder et al (2014) [42]	Blood and malaria parasites	A training set (n=10) with parasites (n=8329) and a validation set (n=6) parasites (n=569)	AI model with (1) thresholding algorithm segments potential parasites, and (2) mathematical feature extraction and classification with SVM
Horning et al (2021) [43]	Blood and malaria parasites	Thick model: Subset of 1452 blood samples with 956,531 parasite objects [54]. Thin model: 798 blood samples with more than 92,000 parasites [55]. Tuning slides: 48 slides	Separate AI models for thin and thick regions: Thick-AI model: (1) potential parasites identified through dynamic thresholding and SVM. (2) CNNs (VGG architecture) classify [54]. Thin AI model: (1) potential parasites detected with a gradient-boosted tree classifier. (2) CNNs for classifying parasite stages [55]
Stegmüller et al (2024) [44]	Cervical cytology and cellular atypia	A stratified 4-fold split approach to partition the 307 slides with 1228 tile-level annotations into training, validation, and test sets; 2 public datasets also used	AI model with (1) CNN (ResNet-50) with self-supervised training (DINO) and then supervised training with cell pasting, and (2) 8 most suspicious tiles used for slide classification with multiple instance learning (CLAM)
Holmström et al (2021) [9]	Cervical cytology and cellular atypia	350 WSIs^i^ were used for training with 16,133 annotations made by a pathologist	AI model with (1) a CNN that segments slide into high- and low-grade atypia, and (2) a threshold that decides slide positivity
Sunny et al (2019) [19]	Oral cytology and cellular atypia	252 atypical and 280 normal cell images annotated (90% for training and 10% for validation)	AI model with (1) cells segmented to single cells, (2) a CNN (Inception V3) used for classification, and (3) cut-offs and SVMs based on percentage and mean score of atypical cells and mean cell score for slide diagnosis
Ghaderinia et al (2024) [46]	Sputum and ferning patterns (inflammation)	650 images (520 training and 130 validation) derived from 70 participants	AI model with (1) a CNN (EfficientNet-B0); and (2) CNN output used to classify sample
Soares et al (2024) [47]	Stool and intestinal parasites (both helminths and protozoans)	51,919 images containing 12,225 annotations of 15 parasite species (ranging from 83 to 3297 per species) [56]	AI model with (1) classification with extracted features and probabilistic SVM, and (2) uncertain objects analyzed with a CNN (Vgg-16) [56]
Lundin et al (2024) [49]	Stool and soil-transmitted helminths	388 samples with 15,058 annotations: *Ascaris lumbricoides* (n=2299), *Trichuris trichiura* (n=2727), hookworm (n=552), and artifacts (n=9480)	AI model with (1) YOLOv2 used to detect potential parasites, (2) a CNN (ResNet50) used for classification, and (3) 1 parasite sufficient for slide positivity
Sahu et al (2024) [50]	Urine and urinalysis	A dataset annotated by a pathologist	AI model with (1) a single CNN (YOLOX) to detect objects, and (2) object counts used to grade slide in tiers of positivity
Meulah et al (2022) [51]	Urine and Schistosoma	Both spiked laboratory samples and 33 field samples [17]	AI model with (1) a CNN segmentation model (U-Net architecture) [17]
Oyibo et al (2022) [52]	Urine and Schistosoma	17,799 annotated *Schistosoma haematobium* eggs in 2997 FOV images; dataset split into 80% training and 20% validation set	AI model with (1) a CNN (DeepLabv3-MobileNetV3), (2) egg-shaped ellipses fitted to segmented regions for counting, and (3) 1 parasite fulfilling criteria sufficient for slide positivity
Meulah et al (2024) [53]	Urine and Schistosoma	17,799 annotated *S. haematobium* eggs in 2997 FOV^j^ images; dataset split into 80% training and 20% validation set [52]	AI model with (1) a CNN (DeepLabv3-MobileNetV3), (2) egg-shaped ellipses fitted to segmented regions for counting, and (3) 1 parasite fulfilling criteria sufficient for slide positivity [52]

a AI: artificial intelligence.

b CBC: complete blood count.

c RBCs: red blood cells.

d WBCs: white blood cells.

e CNNs: convolutional neural networks.

f YOLO: You Only Look Once.

g SVM: support vector machine.

h VGG: Visual Geometry Group.

i WSIs: whole slide images.

j FOV: field of view.

### Study Outcomes

When the study outcomes were mapped, differences were observed in the reference standards, study sizes, and performance metrics used. For the 3 studies investigating CBCs, the Pearson correlation coefficient was compared with high-end analyzers and was above 0.9 for all cells except basophils, where the value ranged from 0.6 to 0.8 in all studies [32-34]. Nine studies used manual microscopy of the same samples as the reference standard and reported results with sensitivity and specificity (for malaria, soil-transmitted helminths, Schistosoma, and cervical cell atypia). Across these 9 studies, all reported a sensitivity and specificity of at least 80% except for 1 with a lower sensitivity of 57% [35] and 3 with lower specificity (75.6%, 78.4%, and 48.9%) [9,40,51]. One study included results with and without human expert verification: human verification of AI model findings increased specificity by 29.5% but conversely led to a sensitivity decrease of 0.9% for malaria detection [36]. Seven studies used reference standards such as polymerase chain reaction (PCR) or histology and included comparisons between AI-supported digital microscopy and manual microscopy; in 4 of these 7 studies, a higher sensitivity but lower specificity was reported for AI-supported digital microscopy. Of the remaining 3 studies, 1 evaluated urinalysis, in which manual analysis had higher sensitivity, specificity, or both across all targets [50], 1 for intestinal parasites where the AI had higher sensitivity and the same specificity [47], and 1 for oral atypia where the AI had both higher sensitivity and specificity [19]. The number of samples included in the diagnostic evaluations ranged from 27 to 2250. Most studies (n=15) achieved a low risk for bias according to QUADAS-2; however, some studies either lacked the information needed to properly evaluate bias or had methodological issues (n=7) (Table 4).

**Table 4.** Results for the included studies.

Study	Sample and target	Human verification	Outcome	Manual microscopy	Number of samples	Reference standard	QUADAS-2^a^
Bachar et al (2021) [32]	Blood and CBC^b^	No	*r*^c^≥0.94 (except basophils=0.6)	NR^d^	679	Hematology analyzer	Low
Gasparin et al (2023) [33]	Blood and CBC	Yes	*r*≥0.94 (except eosinophils/basophils=0.81)	NR	550	Hematology analyzer	Low
Gasparin et al (2022) [34]	Blood and CBC	Yes	*r*≥0.91 (except eosinophils/basophils=0.80)	NR	450	Hematology analyzer	Low
4: Akisin et al (2023) [35]	Blood and Downey cells	No	Se^e^ 57%, Sp^f^ 100%	NR	31	Manual microscopy	Mostly low
Hamid et al (2024) [36]	Blood and malaria	Yes	Se 90.2%, Sp 96.2%	Se 89.3%, Sp 100%	190	PCR^g^	Low
Holmström et al (2020) [38]	Blood and malaria	No	*r*=0.90 for parasite counts	NR	27	PCR	Mostly low
Bae et al (2024) [37]	Blood and malaria	No	Se 95.1%, Sp 91.4%	NR	488	Microscopy and RDTs^h^	Low
Ewnetu et al (2024) [39]	Blood and malaria	Yes	Se 83%‐93.9%, Sp 94%‐97.6%	Se 67%‐69.9%, Sp 97%‐98.7%	1165	PCR	Low
Das et al (2022) [40]	Blood and malaria	No	Se 91.1%, Sp 75.6%	NR	2250	Microscopy	Low
Torres et al (2018) [41]	Blood and malaria	No	Site 1: Se 72%, Sp 85% Site 2: Se 52%, Sp 70%	Site 1: Se 68%, Sp 100% Site 2: Se 42%, Sp 97%	Site 1: 400 Site 2: 300	PCR	Low
Linder et al (2014) [42]	Blood and malaria	Yes	Se 95%, Sp 100%	NR	31	Microscopy	Low
Horning et al (2021) [43]	Blood and malaria	No	Se 86.7%, Sp 100%	NR	35	Microscopy	Low
Stegmüller et al (2024) [44]	Cervical cytology and cellular atypia	No	Mean area under curve 77.5	NR	307 (4-fold split)	Microscopy	Low
Holmström et al (2021) [9]	Cervical cytology and cellular atypia	No	Se 100%, Sp 78.4%	NR	361	Microscopy	Mostly low
Sunny et al (2019) [19]	Oral cytology and cellular atypia	No	Se 89%, Sp 100%	Se 59%, Sp 67%	30	Histology	Low
Ghaderinia et al (2024) [46]	Sputum and ferning patterns (inflammation)	No	Se 94.3%, Sp 95.9%	NR	160	CT^i^	Mostly low
Soares et al (2024) [47]	Stool and intestinal parasites (both helminths and protozoans)	No	Se 86%, Sp 100%	Se 81%, Sp 100%	73	Manual and AI^j^ microscopy	Mostly low
Lundin et al (2024) [49]	Stool and soil-transmitted helminths	No	Se 76.4%‐91.9% Sp 89.7%‐98.2%	NR	792	Microscopy	Low
Sahu et al (2024) [50]	Urine and urinalysis	No	Se ≥81% except for bacteria (76%) and casts (71%), Sp ≥88%	Se ≥94% and Sp ≥93%	240	Microscopy	Mostly low
Meulah et al (2022) [51]	Urine and Schistosoma	No	Se 87.3%, Sp 48.9%	NR	487	Microscopy	Low
Oyibo et al (2022) [52]	Urine and Schistosoma	No	Se 93.8%, Sp 93.9%	NR	65	Microscopy	Mostly low
Meulah et al (2024) [53]	Urine and Schistosoma	No	Se 62.9%, Sp 78.8 %	Se 61.9%, Sp 96.4%	339	PCR and particle lateral flow test	Low

a QUADAS-2: Quality assessment of diagnostic accuracy studies 2.

b CBC: complete blood count.

c *r*: Pearson correlation coefficient.

d NR: Not reported.

e Se: sensitivity.

f Sp: specificity.

g PCR: polymerase chain reaction.

h RDT: rapid diagnostic test.

i CT: computed tomographic scan.

j AI: artificial intelligence.

## Discussion

### Summary

This scoping review included 22 publications deploying AI-supported digital microscopy in PHC laboratories for multiple targets, published in 15 different journals. These studies fulfilled the concepts of using AI and digital microscopy to achieve a slide-level diagnosis in PHC laboratories. The number of included studies was low, given the extensive research on AI in medical imaging. The exclusion of 58 papers due to the absence of sample-level diagnoses and of 71 papers due to not being conducted in PHC laboratories suggests that most research has focused on target detection or advanced laboratory settings, rather than evaluating end-to-end diagnostic systems for PHC use. This is notable, given the potential benefits of such technologies in PHC laboratories. However, 9 of the 22 included studies were published in 2024 indicating an upward trend in studies focused on AI-supported digital microscopy at the PHC level.

The studies targeting specific diseases primarily focused on conditions that disproportionally affect vulnerable populations. The results from the included studies in this scoping review indicate that AI-supported digital microscopy can achieve accuracy comparable to that of standard microscopy for malaria, intestinal parasites, cell atypia, and urinalysis; to that of computed tomography for detecting pulmonary inflammation in patients with COVID-19; and to that of conventional hematology analyzers for CBC. Diagnostic accuracy comparable to the reference standard was defined as sensitivity and specificity of >80% or a Pearson correlation of >0.90. The reported results also indicate that AI-supported digital microscopy could be particularly advantageous for increasing sensitivity, as 6 out of 7 (85.7%) studies comparing it with manual microscopy reported higher sensitivity for AI-supported digital microscopy. Furthermore, the objective of the scoping review was to map target-agnostic challenges and solutions regarding sample preparation, scanning, AI methods, and human integration and discuss future implications for AI-supported digital microscopy in PHC laboratories.

### Sample Preparation

Variability in target morphology and artifacts may reduce AI performance and can be introduced in all steps from sample collection to scanning. Manual steps in sample preparation are prone to introducing variability, and all included studies involved such steps, with 12 relying on entirely manual preparation. Decreased specificity due to sample variability was observed in one study, where poorly prepared samples led to the introduction of artifacts [41], and in another study where synthetically prepared samples in the training dataset lacked artifacts present in real-world samples [51]. Sensitivity can also be affected by variability in preparation, as demonstrated in one study on soil-transmitted helminths [49]. This indicates that variability introduced during sample preparation may be a major hurdle when developing AI-supported digital microscopy for PHC laboratories as more steps are performed manually.

There are possible solutions to sample variability. For example, improving consistency through good laboratory practices and standard operating procedures is one way to minimize sample variability; however, this requires system-specific training for personnel, good laboratory infrastructure, and quality controls, which might reduce the feasibility of implementation in PHC laboratories. The use of equipment such as cartridges to limit manual steps is another approach to minimize variability [33,36,50]. This may lower the demands on personnel; however, using disease-specific consumables may introduce issues, for example, increased costs. Another potential approach to minimize variability is to simplify sample preparation, for example, by removing staining or smearing steps [57]. Although this could reduce variability, it may also lead to a loss of valuable diagnostic information, in turn decreasing the AI model performance.

### Scanning

The scanner needs to capture sufficient information to allow AI model classification of targets, but scanning large sample areas at high magnification is time-consuming. The scanning time can be decreased by analyzing a smaller sample area. However, this can lead to a reduced ability to detect low-density targets, highlighting a trade-off between faster diagnostics and high sensitivity for these cases. This is exemplified by one solution for malaria, where clinicians are able to increase the area analyzed to detect low-density infections [39]. Another solution to decrease scanning time is to use a lower magnification than what is conventionally used by microscopists: this approach achieved diagnostic accuracy comparable to manual microscopy for cytology [19], malaria [36], and parasitic infections [49,53]. Nonetheless, the use of lower magnification could result in information loss that reduces the AI model performance.

### Training Data

All studies that specified the AI-training methods used variants of supervised learning which require annotated data. Annotating data is time-consuming and requires digitized samples that are rarely produced in PHC laboratories due to limited access to scanners. Therefore, many studies had to collect and annotate their own datasets rather than access existing data. One study also used laboratory-enriched samples to increase the number of targets [51]. In some cases, certain targets were underrepresented in the dataset, which caused the AI models to perform poorly on those [32,43], emphasizing the challenges of limited training data. To overcome limited datasets, approaches, such as using data augmentation, publicly available datasets, CNNs with pretrained weights, and unsupervised learning, were deployed [38,44,52]. Although there are many ways to limit the effect of small datasets, the improved diagnostic performance in studies, iteratively collecting larger datasets, highlights that insufficient training data remain a limiting factor when developing AI-supported digital microscopy for PHC laboratories [17,33,34,52]. Larger studies and collaborations that allow data sharing could provide solutions to the issue of limited training data.

### AI Analysis Pipeline

The AI analysis pipelines used can be broadly divided into 3 main steps: FOV identification, FOV classification, and aggregation for sample-level diagnosis (Figure 3). Given that the analysis of a single sample took more than 30 minutes in some studies and that access to graphics processing units may be limited in PHC laboratories, efficiency becomes important. One strategy to minimize computational demands is to combine identification and classification, as implemented in the You Only Look Once framework, which uses a single CNN [34,50,58]. Another strategy is to first identify targets using fast and computationally efficient methods and subsequently feed the suspicious FOVs into more computationally intensive algorithms for classification. Using an initial object identification step may also enhance the uniformity of the data entering the classification stage, which may be particularly beneficial due to the variability in manually prepared samples that are commonly used in PHC laboratories [19].

For the third step, slide-level classification, different approaches were used: slides were classified as positive if a single positive target was detected; others applied cutoffs to reduce noise and false positives. In addition, certain studies used methods such as SVMs [19] or multiple instance learning [44] to aggregate slide-level results. While these methods may improve classification, they carry a risk of overfitting, especially since the number of training samples at the slide level is much smaller than at the object level.

### Manual Verification

One study investigated AI-supported digital microscopy with and without human verification. In the study, human verification was performed on targets initially classified as positive by the AI models, which led to a 0.9% drop in sensitivity but a 29.5% increase in specificity [36]. This demonstrates that, with human intelligence, AI errors can be identified and removed without a substantial loss of sensitivity. This is in line with the high specificity presented in studies using human verification, which all showed specificity of >90% [39,42]. Expanding human verification to include borderline cases classified as negative may also be used to reduce false negatives and increase sensitivity.

### Reported Diagnostic Performance

The reported diagnostic performance of the studies included in the scoping review indicates that AI-supported digital microscopy may achieve comparable diagnostic accuracy in PHC laboratories; however, it is important to account for methodological choices when interpreting the results and due to the heterogeneity in study designs, comparisons between studies and diseases become challenging. One example of methodological choices is that most studies used manual microscopy as the reference standard. Since microscopy itself is an imperfect diagnostic test, it can affect the performance of the index test and may result in over- or underestimation of the diagnostic accuracy of AI-supported microscopy. Two studies argued that this limitation may have reduced the apparent diagnostic accuracy of AI-supported digital microscopy [49,50]. Another aspect to consider is the number of samples on which the method was evaluated. For example, the study in which the AI-supported digital microscopy had higher sensitivity and specificity than manual microscopy analyzed 30 samples [19]. Generally, the QUADAS-2 tool indicated a low risk of bias. However, it did not capture the issue of AI models being trained on samples from the same collection, which is a potential source of poor generalizability for AI. This can occur even when the detection algorithms are trained on different datasets, for example, when thresholds or rules for deriving slide-level diagnoses are developed using the same slides on which diagnostic performance is later evaluated, leading to inflated estimates of diagnostic accuracy. However, some studies avoided training on the data from the same collection, included more than 100 test samples, had low risk of QUADAS-2 bias, and used a more advanced reference standard and still achieved comparable or better results than manual microscopy [33,36,39,53].

### Limitations

A limitation of the extraction process was the lack of consistent terminology used in the field. This was exemplified in the search block aimed at identifying PHC. Terms such as “low-cost” and “PHC” were included but not “remote,” which was used to describe one study that fulfilled the inclusion criteria [59]. Another limitation was the broad definition of PHC laboratories adopted from Fleming et al, which led to the inclusion of studies using relatively advanced methods, such as oil immersion scanning at 100× magnification and specially designed cartridges for sample preparation [2,33,41,42]. These methods may be difficult to implement in some PHC laboratories, but to achieve a more comprehensive overview of the field, the inclusion of these studies was deemed advantageous [25]. A third limitation stems from the lack of standardized methodological descriptions in the included studies. In some cases, key information, such as scanner magnification, was missing or reported inconsistently across studies, which complicated comparisons in the scoping review.

### Steps Needed to Achieve Clinical Implementation

In this scoping review, we identified hurdles that were shared across several studies and that must be overcome before implementing AI-supported digital microscopy. Many developers have recognized a need to iteratively improve their AI-supported digital microscopy; thus, a framework that enables continuous improvements might be advantageous for supporting the development of more accurate AI models. This requires health policy guidelines and frameworks that give details on how these processes should be conducted [60]. Another hurdle in the implementation of AI-supported digital microscopy is cost. The development of lower-cost scanners has reduced expenses; however, most commercially available scanners remain more expensive than traditional microscopes [38,61]. Microscopes typically function reliably over long periods, and scanners may need comparable longevity for AI-supported digital microscopy to be cost-effective. One potential solution to this is modular scanner construction, which may improve its lifespan through component updates and thereby its sustainability. Some systems in this review were developed for specific diseases, which increases the cost of implementing them in PHC laboratories, as multiple systems would have to be acquired to replace microscopes. To make it economically feasible to implement AI-supported digital microscopy, it may, therefore, be necessary to adopt a multipurpose approach where systems are developed for multiple diseases. Some studies show that scanners can digitize different samples and similar approaches can be applied to different diseases [9,37,49]. Systems developed for specific diseases may instead be useful in large screening programs or epidemiological surveys, for example, for soil-transmitted helminths, malaria, or cancer screening. Cost-effectiveness trials could provide guidance for the feasibility of AI-supported digital microscopy and evaluate single-disease systems against multipurpose platforms.

### Potential Implications for PHC Diagnostics

AI-supported digital microscopy has potential advantages compared with manual microscopy in PHC laboratories. First, it could improve diagnostic accuracy, especially sensitivity, and this may be further enhanced by incorporating human verification [39,53]. Second, it might increase the access and timeliness of diagnostics by allowing diagnostic procedures to be performed at the POC in PHC laboratories and eliminate the need to send samples elsewhere for analysis [9,19]. Third, it could alleviate the workload of personnel through task shifting. This could increase the productivity of experts and thereby access to image-based diagnosis [36,39].

Other POC diagnostic technologies, including rapid diagnostic tests (RDTs) and PCR-based methods, provide alternative means of diagnosing some conditions discussed in this scoping review [62,63]. One included study compared AI-supported digital microscopy with another POC-diagnostic method: a malaria study comparing it with RDTs [39]. The AI-supported digital microscopy had higher sensitivity and specificity than RDTs for some malaria species and settings but lower in others. One review proposed that AI-supported digital microscopy holds more promise than RDTs for malaria diagnosis [64]. However, other studies highlight the possibilities of other methods, such as RDTs and PCR, for POC diagnostics [62].

Implementation of AI-supported digital microscopy could strengthen health systems and increase health equity, particularly where resources are limited such as in scarcely populated areas and LMICs. This may be possible since, in addition to comparable diagnostic accuracy to microscopy, slides can be scanned and analyzed within approximately 30 minutes for multiple diseases [34,39,53]. As the process eliminates the need for microscopy expertise on-site, it could enable timely and accurate diagnostics in PHC laboratories that currently lack this capacity: even if manual verification is required, it can be performed remotely. Moreover, by decentralizing diagnostics, it may reduce referrals to higher-tier health care facilities, alleviating their work and minimizing the risk of referral-related dropouts [12].

### Knowledge Gaps and Research Priorities

This scoping review identified evidence of the feasibility of AI-supported digital microscopy for multiple targets in PHC laboratories. Drawing on the evidence mapped here, future research should prioritize studying scalable and robust systems that can be transferred and implemented in new laboratories and settings. Achieving scalability requires research into AI-supported digital microscopy with an end-to-end perspective, where everything from sample preparation, scanning, and AI analysis until the final diagnosis is accounted for and easily reproducible. Adding to this, research that examines how predeveloped AI-based systems are transferred and implemented in new clinical settings would provide valuable insights into real-world robustness, which was done by some of the included studies. To enable this kind of research, large multisite collaborations are important, which could be facilitated by improved health policy guidelines and frameworks, as well as initiatives led by key stakeholders including governments and nongovernmental organizations. Furthermore, important research priorities include assessing cost-effectiveness and exploring perceived barriers to implementation among patients and health care professionals. Finally, the scoping review’s screening process identified additional potential applications for AI-supported digital microscopy, including tuberculosis, other parasitic diseases, respiratory cytology, sperm motility, and sickle cell anemia, which warrant further investigation in PHC settings [65-69].

### Conclusions

This scoping review identified 22 studies deploying AI-supported digital microscopy in PHC laboratories. For multiple diagnostic purposes, AI-supported digital microscopy achieved comparable results to the reference standard and could be particularly advantageous for increasing sensitivity in diagnosis. Further research is needed on challenges such as generalizability, scalability, and cost-effectiveness. Such evidence is critical to stimulate product development, enable regulatory approval, and support reimbursement and adoption by health care authorities. If the methods can be demonstrated to be feasible in real-life clinical PHC settings, translated into medical device products, and carefully integrated into health care systems, they are likely to improve access to diagnostics, particularly in LMICs and scarcely populated regions.

## Supplementary material

10.2196/78500 Multimedia Appendix 1 Search strategy.

10.2196/78500 Multimedia Appendix 2 Original data extraction tool.

10.2196/78500 Multimedia Appendix 3 Quadas-2 tool.

10.2196/78500 Multimedia Appendix 4 Sample collection and scanning.

10.2196/78500 Checklist 1 PRISMA-ScR (Preferred Reporting Items for Systematic reviews and Meta-Analyses extension for Scoping Reviews) checklist.

## Abbreviations

AI: artificial intelligence
CBC: complete blood count
CNN: convolutional neural network
FDA: US Food and Drug Administration
FOV: fields-of-view
LMICs: low- and middle-income countries
PCR: polymerase chain reaction
PHC: primary health care
POC: point of care
PRISMA: Preferred Reporting Items for Systematic reviews and Meta-Analyses
PRISMA-ScR: Preferred Reporting Items for Systematic reviews and Meta-Analyses extension for Scoping Reviews
QUADAS-2: Quality Assessment of Diagnostic Accuracy Studies 2
RDT: rapid diagnostic test
SVM: support vector machine
WHO: World Health Organization
