# Supplementary material for: AI-Supported Digital Microscopy Diagnostics in Primary Health Care Laboratories: Scoping Review
Source: J Med Internet Res. 2026 Jan 5;28:e78500. doi: 10.2196/78500 (PMC12768395; doi:10.2196/78500)
Supplement: Multimedia Appendix 2 [file jmir-v28-e78500-s002.docx]

| Title, author, year | Target disease | Dataset preparation | AI model and training | Quadas-2 risk of bias | Results | Additional comments |
| --- | --- | --- | --- | --- | --- | --- |
|  |  |  |  |  |  |  |
|  |  | How the samples were collected, prepared, and scanned | How the model was trained, the number of samples, the structure of AI |  | Number of samples and endpoints and what it was compared to |  |
