# Supplementary material for: AI-Supported Digital Microscopy Diagnostics in Primary Health Care Laboratories: Scoping Review
Source: J Med Internet Res. 2026 Jan 5;28:e78500. doi: 10.2196/78500 (PMC12768395; doi:10.2196/78500)
Supplement: Multimedia Appendix 4 [file jmir-v28-e78500-s004.docx]

| **Study** | **Sample** | **Target** | **Sample preparation** | **Sample scanning** | **Time for analysis** |
| --- | --- | --- | --- | --- | --- |
| 1: Bachar et al 2021 [25] | Blood | CBC | Cartridge that uses capillary force to create a monolayer of blood, stained with two fluorescent reagents | Sight OLO, scans >200 FOVs with channels for brightfield and fluorescence. No retrievable magnification and resolution | No retrievable information |
| 2: Gasparin et al 2023 [26] | Blood | CBC | Dual-chamber cartridge with blood stained for identification of 1) WBCs and 2) RBCs and platelets | Hilab lens, scans circa 400 FOVs per sample. No retrievable magnification and resolution | Total: 30-40 min |
| 3: Gasparin et al 2022 [27] | Blood | CBC | Dual-chamber cartridge with blood stained for identification of 1) WBCs and 2) RBCs and platelets | Hilab lens, scans circa 400 FOVs per sample. No retrievable magnification and resolution | Total: 30-40 min |
| 4: Akisin et al 2023 [28] | Blood | Downey cells | Manual blood smears stained with May-Grünwald and Giemsa | Mantiscope Slide Scanner, 100x optical magnification with immersion oil | No retrievable information |
| 5: Hamid et al 2024 [29] | Blood | Malaria-parasites | Cartridge that smears 5 μL blood and stains it with Giemsa using hydrogel | Milab, 400 FOVs scanned with circa 200,000 RBCs and a resolution similar to 50x [30] | Total: less than 30 min |
| 6: Holmström et al 2020 [31] | Blood | Malaria-parasites | Manual blood smears stained with DAPI | Custom-built portable digital microscope scanner with brightfield and fluorescence imaging, and a resolution of 0.9 µm | No retrievable information |
| 7: Bae et al 2024 [30] | Blood | Malaria-parasites | Cartridge that smears 5 μL blood and stains it with Giemsa using hydrogel | Milab, 400 FOVs scanned with circa 200,000 RBCs and a resolution similar to 50x [29] | Total: less than 30 min [29,32]; Scanning: 7-10 min |
| 8: Ewnetu et al 2024 [32] | Blood | Malaria-parasites | Cartridge that smears 5 μL blood and stains it with Giemsa using hydrogel | Milab, 400 FOVs scanned with circa 200,000 RBCs and a resolution similar to 50x [29,30] | Total: circa 20 min |
| 9: Das et al 2022 [33] | Blood | Malaria-parasites | Manual blood smears stained with Giemsa | Motic EasyScan Go, 144 FOVs scanned with a 40x (NA 0.75) objective with oil-immersion and z-stacking | Scanning and AI-analysis: 20–30 min |
| 10: Torres et al 2018 [34] | Blood | Malaria-parasites | Manual blood smears stained with Giemsa | Autoscope, 100x oil immersion (NA 1.25) with an optical resolution of circa 0.2 µm, 9 z-stacks | No retrievable information |
| 11: Linder et al 2014 [35] | Blood | Malaria-parasites | Thin blood smears stained with Giemsa | A microscope with a motorized stage, circa 6 mm^2^ with scanned 63x oil immersion (NA 1.4) | No retrievable information |
| 12: Horning et al 2021 [36] | Blood | Malaria-parasites | Manual blood smears stained with Giemsa | Motic EasyScan GO 18 mm^2^ scanned with 40x (NA 0.75), 5 z-stacks | Scanning and AI analysis: 54 min; Scanning: 1) thick 13 min, 2) thin 6 min; AI analysis: 1) thick 29 min 2) thin 7 min |
| 13: Stegmüller et al 2024 [37] | Cervical cytology | Atypical cervical cells | SurePath™ procedure (manual preparation available) and Papanicolaou stain | Grundium Ocus®40 scans with 40x (NA 0.75) in 3 z-stacks | No retrievable information |
| 14: Holmström et al 2021 [7] | Cervical cytology | Cellular atypia | Conventional cytology stained with Papanicolaou | Grundium Ocus®20, scans with 20x (NA 0.4) | Scanning: 5-10 min; uploading 10-40 min |
| 15: Sunny et al 2019 [12] | Oral cytology | Cellular atypia | Manual liquid-based cytology with centrifugation, stained with H&E [38] | Cellscope, scans 100-125 FOVs with 20x (NA 0.4) [38] | AI analysis: 10 min |
| 16: Ghaderinia et al 2024 [39] | Sputum | Ferning patterns (inflammation in COVID-19 patients) | Sputum settled 30 min; 10 μL air-dried on glass slide; no staining | Custom 40x smartphone-compatible mini-microscope; captures the sample in one FOV | No retrievable information |
| 17: Soares et al 2024 [40] | Stool | Intestinal parasites (both helminths and protozoans) | Fecal samples were centrifuged, then processed using flotation and sedimentation [41] | 2000 FOVs scanned per slide. No retrievable magnification and resolution | AI analysis: circa 3 min |
| 18: Lundin et al 2024 [42] | Stool | Soil-transmitted helminths | Kato-Katz thick smears | Grundium Ocus®20, scans with 20x (NA 0.4) | Scanning 5-10; uploading 10-20 min; AI analysis 5 min |
| 19: Sahu et al 2024 [43] | Urine | Urinalysis | Cartridge that concentrates the urine through 5 mins of sedimentation | A custom-made scanner, captures 25 FOVs with a 40x (NA 0.65) and 10x objective in 3 z-stacks | No retrievable information |
| 20: Meulah et al 2022 [44] | Urine | Schistosoma | A membrane capturing particles from 10 mL of urine filtered through it placed on slide | Schistoscope, a custom-made scanner with a 4x objective (NA 0.1) and resolution of 3.3 µm | Scanning: 12 min; AI analysis: 5 min |
| 21: Oyibo et al 2022 [45] | Urine | Schistosoma | A membrane capturing particles from 10 mL of urine filtered through it placed on slide | Schistoscope, a custom-made scanner with a 4x objective (NA 0.1) and resolution of 3.3 µm | Scanning: 12 min; AI analysis: 10-12 min |
| 22: Meulah et al 2024 [46] | Urine | Schistosoma | A membrane capturing particles from 10 mL of urine filtered through it placed on slide | Schistoscope, a custom-made scanner with a 4x objective (NA 0.1) and resolution of 3.3 µm | Scanning and AI-analysis: 25 min |
